# Minimal Contribution of APOBEC3-Induced G-to-A Hypermutation to HIV-1 Recombination and Genetic Variation

**DOI:** 10.1371/journal.ppat.1005646

**Published:** 2016-05-17

**Authors:** Krista A. Delviks-Frankenberry, Olga A. Nikolaitchik, Ryan C. Burdick, Robert J. Gorelick, Brandon F. Keele, Wei-Shau Hu, Vinay K. Pathak

**Affiliations:** 1 Viral Mutation Section, HIV Dynamics and Replication Program, National Cancer Institute at Frederick, Frederick, Maryland, United States of America; 2 Viral Recombination Section, HIV Dynamics and Replication Program, National Cancer Institute at Frederick, Frederick, Maryland, United States of America; 3 AIDS and Cancer Virus Program, Leidos Biomedical Research, Inc., Frederick National Lab, Frederick, Maryland, United States of America; University of Illinois at Chicago College of Medicine, UNITED STATES

## Abstract

Although the predominant effect of host restriction APOBEC3 proteins on HIV-1 infection is to block viral replication, they might inadvertently increase retroviral genetic variation by inducing G-to-A hypermutation. Numerous studies have disagreed on the contribution of hypermutation to viral genetic diversity and evolution. Confounding factors contributing to the debate include the extent of lethal (stop codon) and sublethal hypermutation induced by different APOBEC3 proteins, the inability to distinguish between G-to-A mutations induced by APOBEC3 proteins and error-prone viral replication, the potential impact of hypermutation on the frequency of retroviral recombination, and the extent to which viral recombination occurs in vivo, which can reassort mutations in hypermutated genomes. Here, we determined the effects of hypermutation on the HIV-1 recombination rate and its contribution to genetic variation through recombination to generate progeny genomes containing portions of hypermutated genomes without lethal mutations. We found that hypermutation did not significantly affect the rate of recombination, and recombination between hypermutated and wild-type genomes only increased the viral mutation rate by 3.9 × 10^−5^ mutations/bp/replication cycle in heterozygous virions, which is similar to the HIV-1 mutation rate. Since copackaging of hypermutated and wild-type genomes occurs very rarely in vivo, recombination between hypermutated and wild-type genomes does not significantly contribute to the genetic variation of replicating HIV-1. We also analyzed previously reported hypermutated sequences from infected patients and determined that the frequency of sublethal mutagenesis for A3G and A3F is negligible (4 × 10^−21^ and1 × 10^−11^, respectively) and its contribution to viral mutations is far below mutations generated during error-prone reverse transcription. Taken together, we conclude that the contribution of APOBEC3-induced hypermutation to HIV-1 genetic variation is substantially lower than that from mutations during error-prone replication.

## Author Summary

HIV-1 populations exhibit high levels of genetic variation, allowing the virus to escape immunological and pharmacological selection pressures, and thwarting efforts to develop an effective vaccine; thus, it is critical to understand all sources of HIV-1 genetic diversity. In addition to error-prone reverse transcription and frequent recombination, APOBEC3 proteins, expressed as part of the host intracellular antiviral defense mechanism, could potentially contribute to viral genetic variation through sublethal hypermutation or rescue of hypermutated portions of the genomes without lethal mutations through recombination. Here, we show that hypermutation by APOBEC3 proteins does not affect the rate of recombination; in addition, even in proviruses generated from heterozygous virions containing wild-type and hypermutated genomes, hypermutation only modestly increased the viral mutation rate by twofold. Since the frequency of copackaged wild-type and hypermutated genomes in patients is extremely low, we conclude that hypermutation does not significantly contribute to HIV-1 genetic variation. Analysis of hypermutated sequences from infected patients indicates that the frequency of sublethal mutagenesis is negligible and its contribution to viral variation is substantially lower than that of error-prone viral replication.

## Introduction

Human immunodeficiency virus type 1 (HIV-1) undergoes continuous evolution and adaptation to its host environment resulting in high genetic variation which allows the virus to escape immune responses or to acquire drug resistance [1]. This genetic diversity is generated by three key factors. First, HIV-1, like other retroviruses, has a high mutation rate which has been measured to be between 1.4–3.4 × 10^−5^ mutations/bp/replication cycle [2–6]. This is mainly attributable to error-prone reverse transcriptase (RT) [7] and a minor contribution by RNA polymerase II [3, 8–11]. Second, HIV-1 copackages two viral RNA genomes per virion, allowing recombination during DNA synthesis that reassorts mutations in the copackaged genomes and leads to a further increase in genetic diversity. For HIV-1, recombination rates of ~9 template switches/genome/single cycle have been measured [12, 13]. Third, HIV-1 produces on average 10^11^ virions/day/patient, with ~10^7–^10^8^ productively infected CD4^+^ T cells [1, 14–17], which provides a large population of variants and hence great evolutionary potential.

In 2002, a cellular host protein APOBEC3G (apolipoprotein B mRNA editing enzyme, catalytic polypeptide-like 3G) was identified, which was able to inhibit HIV-1 infection in the absence of the virally-encoded protein Vif (virion infectivity factor) [18]. Some members of the APOBEC3 (A3) family of proteins are potent viral restriction factors and serve as parts of the host’s innate antiviral cellular defense. Among the seven A3 family members, APOBEC3G (A3G), APOBEC3F (A3F), APOBEC3D (A3D), and APOBEC3H (A3H) hapolotypes II, V and VII are packaged into virions in producer cells in the absence of *vif*. These A3s largely contribute to the inactivation of HIV-1Δ*vif* by causing cytidine deamination (cytosine-to-uracil) during reverse transcription of the newly synthesized minus-strand cDNA in the infected target cells [19, 20]. This process results in extensive guanine-to-adenine (G-to-A) mutations in the viral double-stranded DNA genome, called hypermutation [6], which introduces substitutions and stop codons that often lead to the formation of replication-defective proviruses. Lethal mutagenesis of HIV-1 by A3 proteins can be observed in a single round of viral replication [21–23]. In addition, A3 proteins have also been shown to block the HIV-1 life cycle through non-editing mechanisms, as catalytic site mutants are still able to retain some antiviral activity [24, 25]. A3 proteins can inhibit reverse transcription by blocking RT template binding, reducing tRNA binding and processing, inhibiting strand transfer events, and blocking cDNA synthesis elongation [24–33]. Furthermore, A3G and A3F have been shown to inhibit integration by interfering with tRNA primer removal or blocking the 3’ processing of viral DNA ends by integrase, respectively [32, 34]. HIV-1 has evolved to protect itself against A3 proteins by expressing Vif, which targets the A3 proteins for degradation through the ubiquitin-proteasome pathway [35–41]. This in turn excludes the A3 proteins from being encapsidated into viral particles, leading to a productive viral infection.

Analysis of patient proviral sequences has shown the presence of G-to-A hypermutation, indicating that Vif is not always completely successful at degrading the A3 proteins. Depending upon the study, ~25% (9–43%) of patient-derived proviral sequences are hypermutated [42–46]. A3 proteins are characterized by their preference to introduce cytidine deamination in certain dinucleotide motifs: 5’GG→AG for A3G and 5’GA→AA for A3F, A3D, or A3H [21, 47–54]. Although G-to-A mutations in hypermutated proviral sequences found in patients exhibit specific sequence contexts, these analyses are complicated by mutations introduced by RT. Studies of RT fidelity *ex vivo* have identified that one of the major substitutions introduced by RT is also G-to-A; however the sequence contexts in which these mutations occur have not been fully defined [2–6, 55–57].

Currently, the extent to which hypermutated genomes in HIV-1 infected patients contribute to viral genetic variation and evolution is being debated and has not been clearly determined. It has been proposed that A3 sublethal mutagenesis has the potential to contribute to viral variation and the emergence of drug resistance mutations [44, 58–67]. However other studies have proposed that A3-induced hypermutation is an “all or nothing” phenomenon [64], failed to find a correlation between hypermutation and emergence of drug resistance mutations [60], and observed purifying selection such that viral RNA incorporated into the virion will contain little to no hypermutation [68].

Therefore, the role of hypermutation on the HIV-1 life cycle and its contribution to genetic diversity are currently unclear. To address these important questions, we determined the effect of hypermutation on recombination and the contribution of hypermutation to the HIV-1 mutation rate per replication cycle. These studies clarify the role of APOBEC3 proteins in HIV genetic variation and significantly contribute to the resolution of a long-debated question in HIV biology.

## Results

### HIV-1 constructs containing A3G- and A3F-induced hypermutation

**Fig 1:**
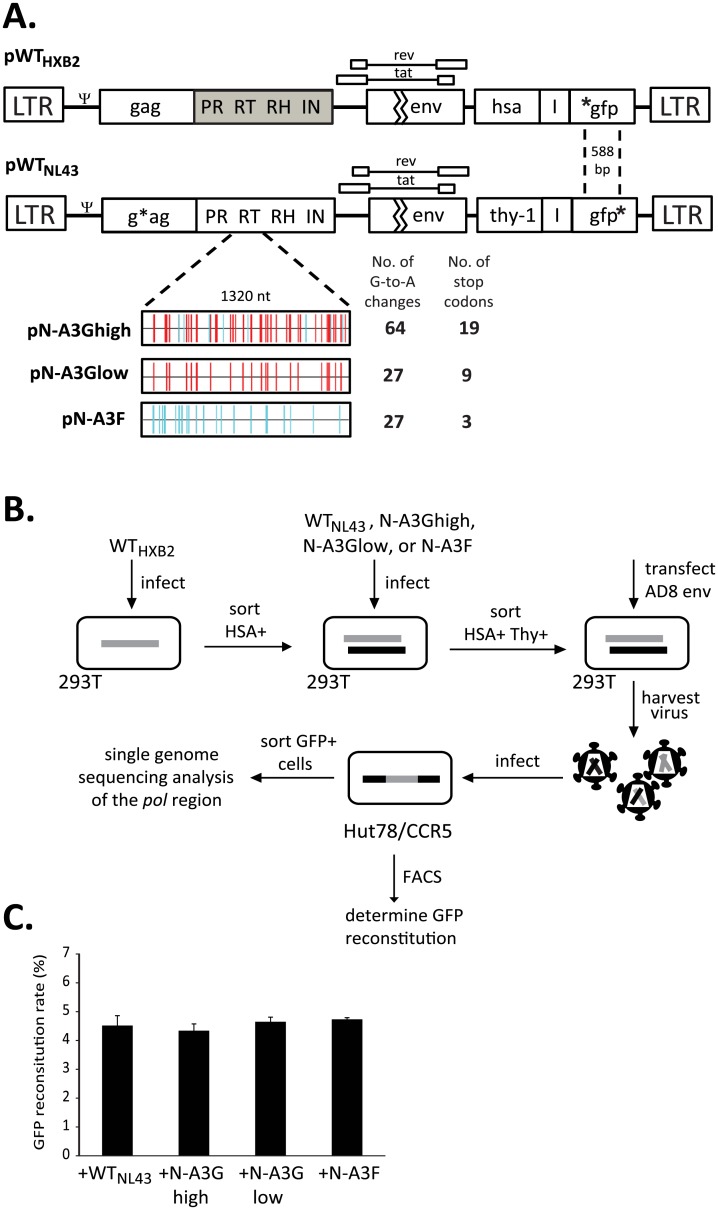
Experimental system to study the contribution of hypermutation to HIV-1 recombination and genetic variation. (A) Plasmids pWT_HXB2_ and pWT_NL43_ are HIV-1-based constructs expressing *hsa*-IRES-*gfp* or *Thy*-IRES-*gfp*, respectively, from the *nef* open reading frame. For pWT_HXB2_, the *pol* region is replaced with HXB2 *pol* sequence (gray shaded region); *gfp* in the nef open reading frame contains an inactivating mutation at the 5’ end of *gfp* (*gfp). For pWT_NL43_, the *pol* region remains NL4-3 and *gfp* in the nef open reading frame contains an inactivating mutation at the 3’ end of *gfp* (gfp*). The inactivating mutations in *gfp* are 588 bp apart. All constructs contain inactivating mutations in *vif*, *vpu*, *vpr* and *env*. * denotes inactivating mutations in *gfp* and *gag* genes. Constructs derived from pWT_NL43_ contain a modified RT region hypermutated by A3G (pN-A3Ghigh or pN-A3Glow), or by A3F (pN-A3F). The number of G-to-A changes relative to pWT_NL43_ and number G-to-A changes leading to a stop codon is shown on the right. Red vertical lines indicate GG→AG mutations, while blue vertical lines indicate GA→AA mutations (Hypermut [102]; www.hiv.lanl.gov). (B) Single cycle recombination system to study the effects of hypermutation. Briefly, 293T cells were infected with WT_HXB2_-dervied viruses at a low MOI, and HSA^+^ cells were enriched by sorting. These HSA^+^ cells were then infected at a low MOI with virus derived from either WT_NL43_, N-A3Ghigh, N-A3Glow, or N-A3F and sorted for HSA^+^/Thy^+^ cells. The respective four cell lines were then transfected with a plasmid expressing HIV-1 Env from the AD8 strain; virus was harvested and used to infect Hut78/CCR5 target cells. The infected cells were used for flow cytometry analysis or sorted for GFP^+^ cells to be used in single genome sequencing analysis. (C) Rate of GFP reconstitution for each of the four vector pairs after infection into Hut78/CCR5 target cells. Average of 2 independent infections. Paired two-sample *t*-tests of comparison of each group to WT indicated no significant differences (*P* > 0.5).

To study the effects of hypermutation on HIV-1 recombination and mutation rates per replication cycle, we generated five HIV-1 constructs (Fig 1A). These constructs are based on HIV-1 NL4-3 (GenBank AF324493.2) and contain the necessary *cis*-acting elements required for virus expression and replication. They also contain *gag* and *pol* genes, and express functional Tat and Rev, but contain inactivating deletions in *vif*, *vpu*, *vpr*, and *env*. In the construct pWT_HXB2_, the NL4-3 *pol* sequence has been replaced with the *pol* sequence from the HXB2 isolate; in addition, it also expresses from the *nef* open reading frame, the mouse heat stable antigen (*hsa*) followed by the internal ribosomal entry site (IRES) from encephalomyocarditis virus and green fluorescent protein gene (*gfp*) with an inactivating mutation at the 5’ end of the gene.

The remaining four constructs contain the NL4-3 *pol* sequences: either completely wild type (pWT_NL43_), or a *pol* sequence in which the RT region contains 64 G-to-A changes (pN-A3Ghigh) or 27 G-to-A changes (pN-A3Glow) at A3G target sites, or contains 27 G-to-A changes (pN-A3F) at A3F target sites. These G-to-A changes are relative to pWT_NL43_, and the 64 G-to-A changes are high while 27 mutations are low, relative to an average of 42 G-to-A mutations estimated in the same RT region of hypermutated proviral sequences from patients [69]; analysis of hypermutated proviral sequences used to estimate the number of G-to-A mutations in the RT region is discussed later in Results Section. The 64, 27, and 27 G-to-A changes in pN-A3Ghigh, pN-A3Glow, and pN-A3F resulted in 19, 9, and 3 stop codon mutations, respectively. In addition, these four constructs contain in *nef* a mouse CD90.2 gene (*thy*) followed by IRES and *gfp* with an inactivating mutation at the 3’ end of the gene. All *thy*-containing constructs also contain a frameshift mutation resulting in a premature stop codon in *gag* to prevent expression of the Gag/Gag-Pol polyproteins; hence, functional Gag and Gag-Pol are only produced from WT_HXB2_.

### Creation of 293T producer cells expressing wild-type and hypermutated proviruses to determine effect of hypermutation on recombination

To study recombination, we generated four producer 293T cell lines, each containing a WT_HXB2_ provirus and either WT_NL43_, N-A3Ghigh, N-A3Glow or N-A3F provirus (Fig 1B). These cell lines were generated by sequential infections at low multiplicity of infection (MOI) followed by multiple rounds of cell sorting to ensure that the majority of cells in each cell line contained a single integrated copy of each parental provirus. More than 97% of the cells in the producer cell lines expressed both HSA and Thy markers, with each cell line representing a pool of at least 115,000 independent infection events.

None of the aforementioned constructs express functional HIV-1 Env; to produce infectious viruses, we transfected the four cell lines (WT_HXB2_/WT_NL43_, WT_HXB2_/N-A3Ghigh, WT_HXB2_/N-A3Glow, and WT_HXB2_/N-A3F) with a plasmid expressing CCR5-tropic HIV-1 envelope from the AD8 strain [70]. Since 293T cells lack the CD4 receptor, the viruses containing the AD8 Env cannot reinfect the producer cells. We then used the resulting AD8 Env containing viruses to infect Hut78/CCR5 target cells at a low MOI (<0.08) in order to minimize the chance of double infection.

In the producer cells, the full-length RNAs from the two parental proviruses can be assorted randomly prior to being packaged into virions [71–73], resulting in the formation of homozygous virions (both RNAs from same parent) and heterozygous virions (two RNAs from different parents). During DNA synthesis, RT can switch templates between the copackaged RNAs to generate recombinants containing portions of both genomes [74, 75]. Although recombination can occur in all viruses, the two RNAs in the homozygous viruses contain the same inactivating mutation in *gfp*, and the resulting DNA will have a mutant *gfp*. In contrast, the two RNAs in the heterozygous viruses have different mutations in *gfp*, and recombination between the two mutations can reconstitute a functional *gfp* that confers a GFP^+^ phenotype to the target cell. Therefore, expression of a functional GFP in target cells can be used to identify proviruses derived from heterozygous virions.

### Effect of hypermutation on the recombination rate

The HIV-1 constructs used in this system express either *hsa* or *thy*; thus target cells infected with these viruses would be HSA^+^ or Thy^+^. However, only recombinant proviruses generated from heterozygous particles can reconstitute a functional *gfp* and confer the GFP^+^ phenotype. Therefore, we used the frequency of the *gfp* reconstitution as a measurement for HIV-1 recombination rate. Flow cytometry analysis of the infected target Hut78/CCR5 population showed that the recombination rate, as measured by the reconstitution of *gfp*, was not significantly different for all four vector pairs: 4.5%, 4.3%, 4.6% and 4.7% for WT_HXB2_/WT_NL43_, WT_HXB2_/N-A3Ghigh, WT_HXB2_/N-A3Glow, and WT_HXB2_/N-A3F, respectively (*p* > 0.05; one-way ANOVA and paired two-sample *t*-tests) (Fig 1C). This result indicated that the presence of hypermutation in *pol* did not affect the frequency of recombination in *gfp*. Only the proviruses derived from heterozygous viruses can confer GFP^+^ in target cells; thus, these results also indicated that hypermutation in *pol* did not affect the copackaging efficiency of RNAs from two parental proviruses into the same viral particle.

### Single genome sequencing of retroviral recombinants and determination of recombination junctions

**Fig 2:**
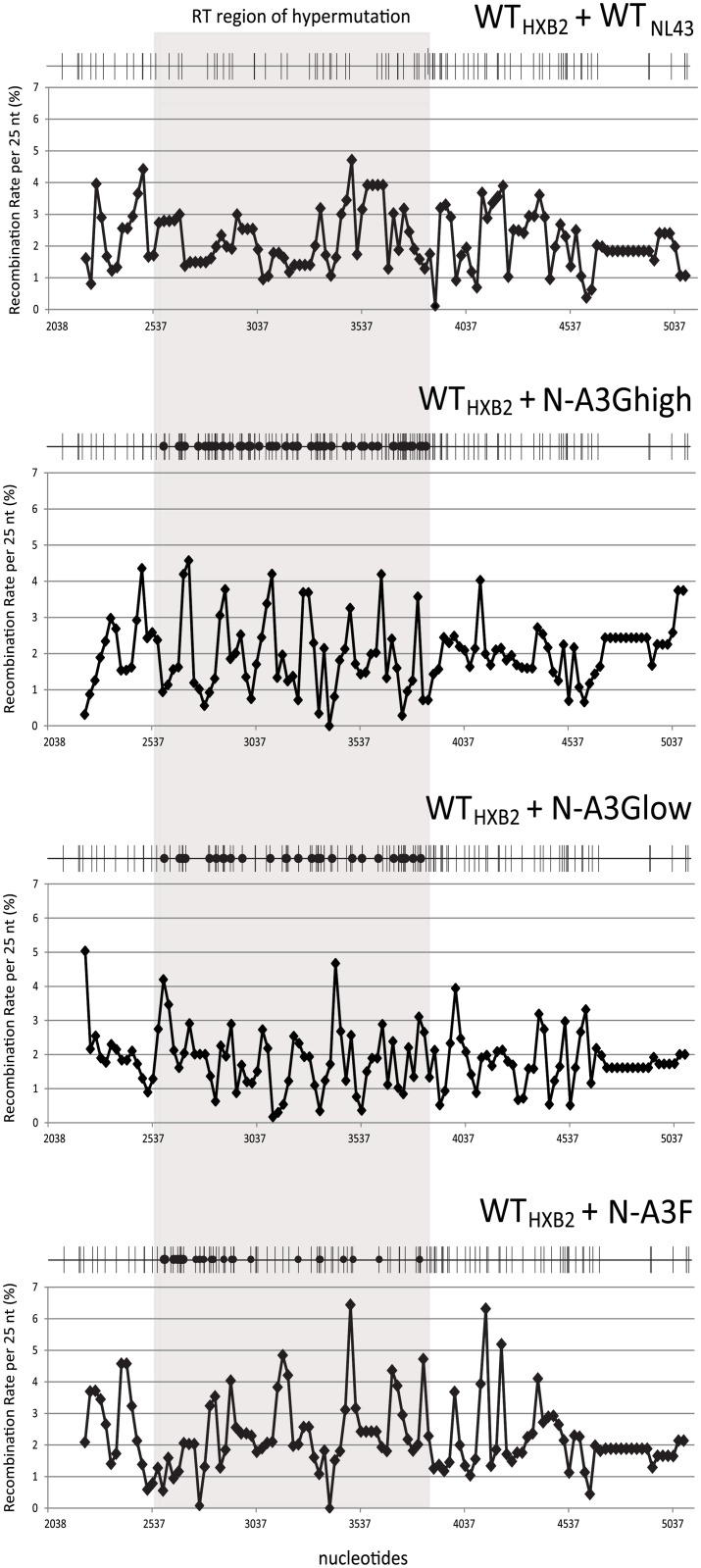
Single-cycle recombination rates across the target *pol* gene. The recombination rate per 25-nt region is plotted for each pair of parent viruses. The RT region where hypermutation was introduced is shaded in gray. Nucleotide numbering corresponds to HXB2 (GenBank Accession number K03455). A total of 96, 154, 121, and 121 polymorphic sites are shown as black vertical lines between HXB2 and N-A3Ghigh, N-A3Glow, and N-A3F, respectively, whereas black circles indicate positions of G-to-A hypermutation in the RT region (Highlighter for Nucleotide Sequences v2.2.3 [103]: www.hiv.lanl.gov).

To further analyze recombination events that occurred between wild-type and hypermutated sequences, we harvested viruses from the aforementioned four producer cell lines and infected target Hut/CCR5 cells in two sets of independent experiments. GFP^+^ cells, which were infected with heterozygous virions, were enriched through multiple rounds of cell sorting until >87% of cells were expressing GFP. Two pools of GFP^+^ cells were generated for each pair of parental viruses and each target cell pool contained at least 7,700 individual infection events that yielded GFP^+^ cells. Genomic DNA from GFP^+^ cell pools was isolated and subjected to single-genome sequencing (SGS) for the *pol* region, and the results from both GFP^+^ pools were combined. A total of 152, 140, 141, and 132 single genome sequences were recovered from WT_HXB2_/WT_NL43_, WT_HXB2_/N-A3Ghigh, WT_HXB2_/N-A3Glow, and WT_HXB2_/N-A3F samples, respectively. Within the 3096-nt stretch of *pol* that was sequenced, there are 96 distinct polymorphisms present between WT_HXB2_ and WT_NL43_, resulting in 97% nucleotide sequence identity. Within the 1320-nt RT region there are 37 distinct polymorphisms present between WT_HXB2_ and WT_NL43_ (97% sequence identity); due to the introduction of hypermutations in RT, there are 95, 62, and 62 polymorphisms present in RT between WT_HXB2_ and N-A3Ghigh, N-A3Glow and N-A3F, resulting in 92%, 95% and 95% sequence identity, respectively (Fig 2; see polymorphic site distribution).

Using polymorphic sites as reference points and the sequences obtained from SGS, we determined the recombination junctions in the entire *pol* for each progeny recombinant. The average distance between marker sites in *pol* for WT_HXB2_/WT_NL43_, WT_HXB2_/N-A3Ghigh, WT_HXB2_/N-A3Glow, and WT_HXB2_/N-A3F is 31, 19, 24 and 24 nucleotides, respectively. At these marker distances, the probability of unobserved double-crossover events is extremely low. Most of the 565 recombinants recovered had a unique recombination pattern. The average number of crossovers per clone in *pol* (3 kb) was determined to be 2.4, 2.3, 2.1 and 2.7 for WT_HXB2_/WT_NL43_, WT_HXB2_/N-A3Ghigh, WT_HXB2_/N-A3Glow, and WT_HXB2_/N-A3F, respectively (Table 1). In comparison to WT_HXB2_/WT_NL43_, there were no significant differences in the numbers of crossovers in *pol* when one of the parental viruses had hypermutations (all *p* values > 0.05; Wilcoxon rank sum test). Further analysis of only the RT region where hypermutation was introduced, the average numbers of crossovers per clone was (1.3 kb) were 1.1, 1.0, 1.0 and 1.2 for WT_HXB2_/WT_NL43_, WT_HXB2_/N-A3Ghigh, WT_HXB2_/N-A3Glow, and WT_HXB2_/N-A3F, respectively (Table 2). These numbers were not significantly different from WT_HXB2_/WT_NL43_ or from each other (all *p* values > 0.05; Wilcoxon rank sum test), nor were they significantly different from the numbers of crossovers in a region of similar size from RNase H to integrase (1.2 kb) that was not hypermutated in comparison to WT_HXB2_/WT_NL43_: 1.0, 1.0, 0.9 and 1.0 for WT_HXB2_/WT_NL43_, WT_HXB2_/N-A3Ghigh, WT_HXB2_/N-A3Glow, and WT_HXB2_/N-A3F, respectively (all *p* values > 0.05; Wilcoxon rank sum test). Additionally, in all four vector pairs the 0.4-kb protease region also contained similar numbers of crossovers per clone in comparison to WT_HXB2_/WT_NL43_: 0.4, 0.3, 0.3, and 0.4 for, WT_HXB2_/WT_NL43_, WT_HXB2_/N-A3Ghigh, WT_HXB2_/N-A3Glow, and WT_HXB2_/N-A3F, respectively (all *p* values > 0.05; Wilcoxon rank sum test) (Table 2). Thus, hypermutation did not affect the average number of crossovers in a single replication cycle.

**Table 1:** Distribution and average number of crossovers in *pol* observed in recombinants.

	No. of Recombinants			
	WT_HXB2 +_	WT_HXB2 +_	WT_HXB2 +_	WT_HXB2 +_
No. of crossovers	WT_NL43_	N-A3Ghigh	N-A3Glow	N-A3F
0	19	19	24	16
1	35	29	32	30
2	36	39	37	25
3	31	25	23	22
4	14	9	9	15
5	5	9	7	12
6	5	6	7	6
7	2	3	2	3
8	3	1	0	1
9	1	0	0	0
10	0	0	0	2
11	1	0	0	0
Total no. clones	152	140	141	132
Avg. no. of				
crossovers per clone*	2.4	2.3	2.1	2.7

*The average number of crossovers per clone was calculated as follows: sum of [(number of crossovers) × (number of recombinants with that many crossovers)]/ total number of recombinant clones.

**Table 2:** Crossover analysis for recombinants throughout *pol*.

Vector pair	Avg. no. of crossovers in *pol* (PR/RT/RNaseH/IN)	Avg. no. of crossovers in PR	Avg. no. of crossovers in RT	Avg. no. of Crossovers in RNaseH + IN
WT_HXB2 +_ WT_NL43_	2.4	0.35	1.07	1.01
WT_HXB2 +_ N-A3Ghigh	2.3	0.32	0.99	1.03
WT_HXB2 +_ N-A3Glow	2.1	0.30	0.96	0.88
WT_HXB2 +_ N-A3F	2.7	0.39	1.23	1.04

To further characterize the distribution of crossovers for each pair of viruses, we determined the number of recombination events in each region between two neighboring polymorphic sites. We then calculated the recombination rate/nucleotide/genome by dividing the observed events by the number of nucleotides between the two polymorphic sites and then by the number of genomes sequenced. Using the recombination rate of each nucleotide, we summed the rates for 25 nucleotides to generate the recombination rates per 25-nt segment. The results of these analyses are summarized in Fig 2 and the RT regions containing the hypermutations are shaded in grey. These results showed that recombination events can be observed throughout the *pol* gene; furthermore, crossovers occur throughout the RT regions regardless of the presence of hypermutations in the RT-coding region of one of the parents. Taken together, our results showed that once a genome was packaged into the virion, the presence of hypermutations in the RNA did not affect the frequency or the distribution of the crossovers events.

### Contribution of hypermutation to the HIV-1 mutation rate

**Fig 3:**
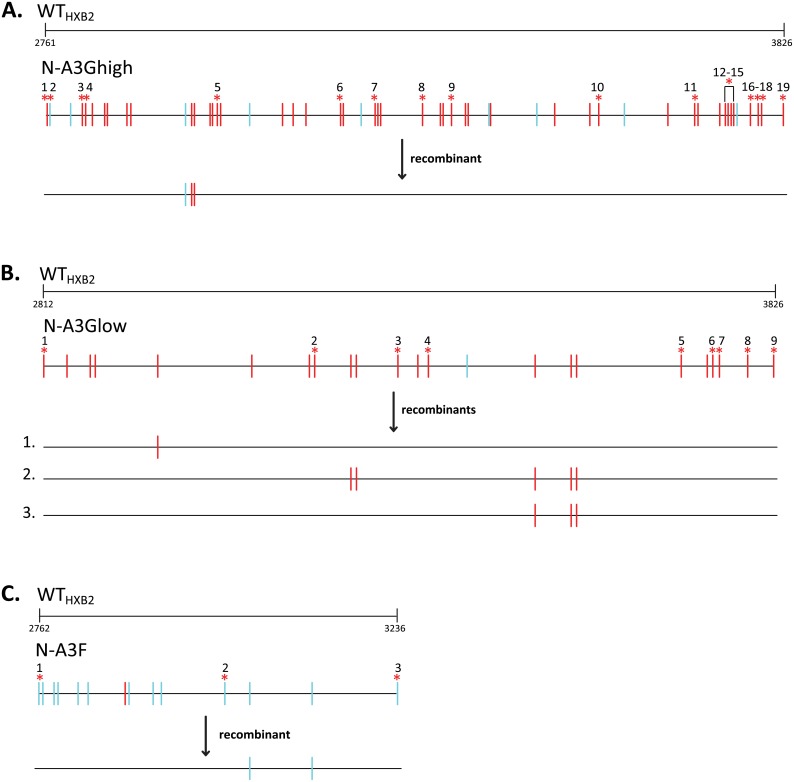
Recombinants with G-to-A changes after single-cycle recombination assay. Distribution of G-to-A changes (relative to WT_HXB2_) for parental N-A3Ghigh (A), N-A3Glow (B), and N-A3F (C) pairs, and for resulting recombinants with non-lethal G-to-A changes. Shown are the G-to-A changes retained by each of the recombinants: one recombinant from WT_HXB2_/N-A3Ghigh, three recombinants from WT_HXB2_/N-A3Glow, and one recombinant from WT_HXB2_/N-A3F. A total of 14 G-to-A changes were observed in these 5 recombinants. G-to-A changes in the GG→AG and GA→AA dinucleotide context are shown as red and blue vertical lines, respectively (Hypermut [102]; www.hiv.lanl.gov), while lethal G-to-A changes that introduce stop codons are marked by * and numbered. Nucleotide numbering corresponds to HXB2 (GenBank Accession number K03455).

G-to-A substitutions generated by A3 proteins frequently introduce stop codons that lead to loss of expression or expression of aberrant viral proteins resulting in replication defects. Hypermutated sequences in vectors N-A3Ghigh, N-A3Glow and N-A3F contained 19, 9 and 3 stop codons, respectively, due to G-to-A hypermutations (Fig 1A). To determine if any recombinants during template switching acquired G-to-A changes, but not the “lethal” stop codons, we analyzed all recombinants for the presence of G-to-A changes between the first and last stop-codon mutations. Fig 3 depicts the G-to-A changes present between the 19, 9 and 3 stop codons for N-A3Ghigh (Fig 3A), N-A3Glow (Fig 3B) and N-A3F (Fig 3C), respectively. For WT_HXB2_/N-A3Ghigh, we recovered one out of 140 recombinants that acquired three G-to-A changes, but lacked stop codons (Table 3). For WT_HXB2_/N-A3Glow, three out of 141 recombinants acquired one, five and three G-to-A changes, respectively, and for WT_HXB2_/N-A3F one out of 132 recombinants acquired two G-to-A changes without stop codons. Therefore, out of a total of 413 recombinants analyzed (354,642 nts sequenced), 14 G-to-A mutations were rescued from hypermutated genomes without stop codons, and the overall contribution of hypermutation to the HIV-1 mutation rate was 3.9 × 10^−5^ mutations/bp/replication cycle. The mutation rate was slightly lower for the WT_HXB2_/N-A3Ghigh population (2.0 × 10^−5^/bp/replication cycle), and higher for the WT_HXB2_/N-A3Glow population (6.3 × 10^−5^/bp/replication cycle). Thus, the contribution of hypermutation to the HIV-1 mutation rate for a population of heterozygous virions containing a hypermutated genome and a nonhypermutated genome was similar to the retroviral mutation rate of 3.4 × 10^−5^/bp/replication cycle [2].

**Table 3:** Contribution of hypermutation to the HIV-1 mutation rate per replication cycle.

	No. of clones	No. of G-to-A mutations rescued	Total no. of nt sequenced	Mutations/bp/ replication cycle
N-A3Ghigh	140	3	149100	2.0 × 10^−5^
N-A3Glow	141	9	142974	6.3 × 10^−5^
N-A3F	132	2	62568	3.2 × 10^−5^
Total	413	14	354,642	-
Average	-	-	-	3.9 × 10^−5^

### *In silico* modeling of the contribution of A3 hypermutation and recombination to HIV-1 mutation rate

We simulated the potential contribution of A3G- and A3F-mediated hypermutation to viral diversity using a custom in-house MATLAB computer program. The NL4-3 genome was used as the baseline sequence, and the input variables were 1) the number of template switches, 2) the number of G-to-A mutations, and 3) the number of heterozygous virions containing a wild-type and a hypermutated genome simulated to undergo one cycle of replication. Our program randomly selected the locations of the template switches and the locations of the G-to-A mutations. For A3G, we simulated 90% of the mutations at GG sites and 10% at GA sites, and for A3F we simulated 86% of the mutations at GA sites and 14% at GG sites; these ratios were based on our previously published [68] observations of the G-to-A mutations induced by A3G and A3F in *ex vivo* experiments. NL4-3 has 616 GG sites, of which 119 (19.3%) are predicted to generate stop codons upon mutation to AG. NL4-3 also has 756 GA sites, of which 37 (4.8%) are predicted to generate stop codons upon mutation to AA. The program then determined the number of viable recombinants, which was defined by the absence of stop codons, and the mutation rate was then calculated as the number of mutations in nonlethal recombinants divided by the total number of nucleotides.

**Fig 4:**
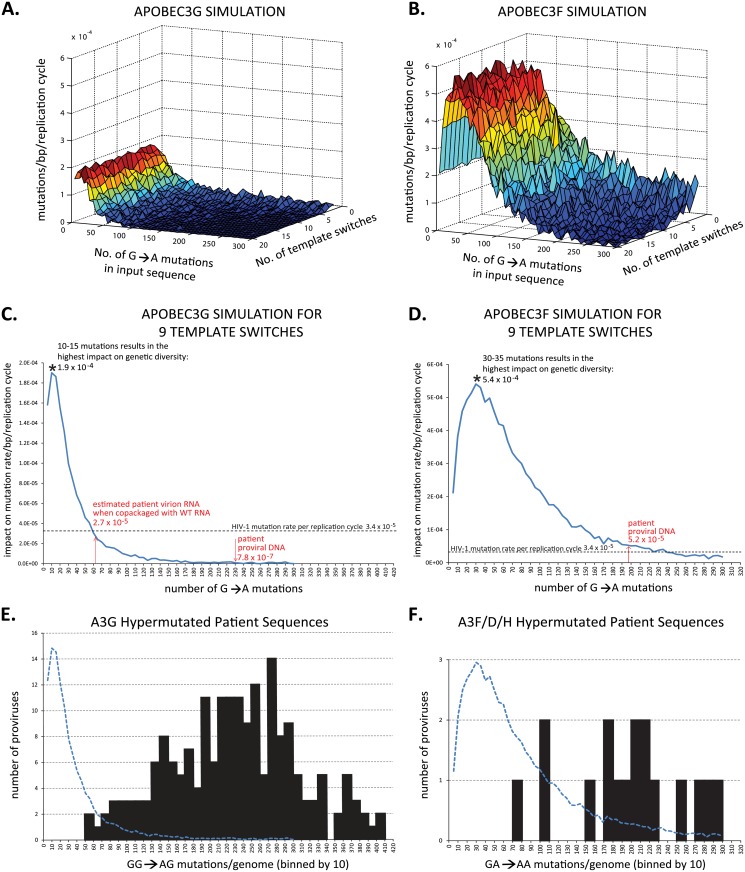
*In silico* modeling of the effect of hypermutation on the HIV-1 mutation rate. (A) and (B) represent the estimated contribution by A3G and A3F to the HIV-1 mutation rate, respectively. The number of G-to-A changes and the number of template switches were varied. One thousand simulations of a heterozygous virion containing a wild-type genome and a hypermutated genome undergoing one cycle of replication were scored per scenario. (C) and (D) show the contribution of GG→AG and GA→AA hypermutations, respectively, to the mutation rate/bp/replication cycle obtained using simulations for 9 template switches/replication cycle. Ten thousand simulations of a heterozygous virion containing a wild-type genome and a hypermutated genome undergoing one cycle of replication were scored per scenario for A3G and A3F. Dotted line indicates the HIV-1 mutation rate of 3.4 × 10^−5^/bp/replication cycle. Shown on the graph in (C) is the estimated 231 G-to-A changes per patient proviral DNA resulting in a mutation rate of 7.8 × 10^−7^/bp/replication cycle and the 62 G-to-A changes per virion RNA genome introduced by A3G (with a HIV-1Δvif), resulting in a mutation rate of 2.7 × 10^−5^/bp/replication cycle. Shown in the graph in (D) is the estimated 197 G-to-A changes per patient proviral DNA introduced by A3F, resulting in a mutation rate of 5.2 × 10^−5^/bp/replication cycle. (E) and (F) distribution of patient proviruses predominantly mutated at GG sites by A3G or GA sites by A3F, A3D, or A3H, respectively. The numbers of G-to-A mutations for the proviral genomes were estimated as described in Tables A and B in S1 File, and plotted in bins of 10 mutations. The simulated impact of hypermutation and recombination on the retroviral mutation rate plotted in C and D (blue line) is superimposed as a blue dotted line in E and F, respectively, for comparison to the distribution of G-to-A mutations per genome.

For both A3G and A3F, the contribution of hypermutation to the HIV-1 mutation rate was independent of the number of template switching events in the simulation, regardless of the starting number of G-to-A changes in the input sequence (Fig 4A and 4B). Thus, using 9 template switches as the average number per single replication cycle [12, 13] and with simulations of 10,000 heterozygous virions containing a wild type and a hypermutated genome undergoing one cycle of replication per scenario, our results showed that when the HIV-1 genome contains 10–15 G-to-A mutations, A3G has the most impact on viral genetic diversity with a mutation rate of 1.9 × 10^−4^ mutations/bp/simulated replication cycle (Fig 4C). However, as the shown in the patient sequence analyses (Fig 4E), none of the 194 hypermutated proviruses that were predominantly mutated at GG sites had the optimal 10–15 GG-to-AG mutations. Instead, hypermutated sequences from patients had an average of 231 GG-to-AG mutations per proviral genome (Table 4 and Tables A and C in S1 File), which is predicted to contribute only 7.8 × 10^−7^ mutations/bp/cycle, a rate that is >200-fold less than the retroviral mutation rate. One caveat to the estimation above is that purifying selection has been observed previously [68] and HIV-1 RNAs packaged into viral particles contain fewer G-to-A mutations (~27%) than those in hypermutated proviral DNA. Therefore, the virion RNA should have an average of 62 G-to-A mutations/genome (27% of 231). As the distribution of the GG-to-AG hypermutations in proviral genomes shows only three of 194 proviruses (~2%) had <80 mutations, suggesting that very few of the proviral genomes would generate an RNA that will be packaged into virions. For the few mutated RNAs that were able to be packaged into viral particle along with a wild-type RNA, our modeling results predicted that with an average of 62 G-to-A mutations/genome, rescue of GG-to-AG mutations through recombination would result in a mutation rate of 2.7 × 10^−5^/bp/cycle, which is similar to the HIV-1 mutation rate/bp/cycle (3.4 × 10^−5^/bp/cycle). Therefore, even in the best case scenario, recombination and rescue of hypermutated portions of proviral genomes without stop codons would only increase the viral mutation rate by twofold. However, since the frequency of copackaging of hypermutated and wild-type genomes is extremely low ([44, 45, 76, 77]; see Discussion), the overall contribution of hypermutation to the viral mutation rate is far less than the mutations generated during reverse transcription.

**Table 4:** Summary of analysis to determine sub-lethal mutagenesis probability for patient sequences that were predominantly hypermutated at GG sites^1^.

Study	No. of Seqs.	Seq.	Total nt	GG→AG Mtns/ genome^2^		GA→AA Mtns/ genome		G→A mtns/ genome		Probability of
				Total	Stops	Total	Stops	Total	Stops	0 Stops^3^
Eyzaguirre et al. 2013	10	Full-length	92,440	206	40	38	2	244	42	6 × 10^−19^
Gandhi et al. 2008	161	Env/nef	73,698	238	46	38	2	276	48	1 × 10^−21^
Ho et al. 2013	23	Gag, Env, nef-LTR	54,550	195	38	33	2	228	40	4 × 10^−18^
Total	194		220,688	44,863	8,659	7,270	349	52,076	9,068	
Average^4^	-		-	231	45	37	2	268	47	4 × 10^−21^

^1^ Details of the analysis of sequences are described in Tables A and C in S1 File.

^2^ Mutations/genome were estimated based on sequence length and average frequency of mutations in the same region of the genome in 11 full-length hypermutated sequences from the Eyzaguirre et al. study [69] to adjust the mutation frequencies for the previously described 5’-to-3’ hypermutation gradient [49, 79–81].

^3^ The probability of sub-lethal mutagenesis was determined by using the average number of stop codon mutations/genome and Poisson distribution.

^4^ The average number of mutations per genome was determined by summing up the total number of mutations estimated per genome for all sequences and dividing by the number of sequences.

We performed a similar analysis for patient proviral genomes that were predominantly hypermutated at GA sites, and were likely hypermutated by A3F, A3D or A3H (Table 5). Proviruses that were predominantly hypermutated at GA sites (17 total) had an average of 197 GA-to-AA mutations/genome. GA-to-AA mutations generate stop codons fourfold less frequently (4.8%) than GG-to-AG mutations (19.3%), suggesting that there will be less purifying selection of GA mutations and more GA-to-AA mutations will be present in the virion RNA. When there are an optimal number of GA-to-AA mutations (30-35/genome; Fig 4F, and assuming these RNAs are copackaged with a wild-type RNA, rescue of GA-to-AA mutations without stop codons through recombination would increase the retroviral mutation rate by 16-fold (5.4 × 10^−4^/bp/cycle). However, the distribution of GA-to-AA mutations/genome in patients (Fig 4F) suggests that none of the hypermutated genomes had an optimal number of mutations (30–35; Fig 4D). The average 197 GA-to-AA mutations/genome is predicted to contribute 5 × 10^−5^ mutations/bp/replication cycle (Fig 4D), which is similar to the retroviral mutation rate (3.4 × 10^−5^/bp/cycle). It is worth noting that the simulations above are based on copackaging of the mutated RNA with wild-type RNA. However, since the frequency of copackaging wild-type and hypermutated genomes is extremely low [44, 45, 76, 77] we conclude that A3F-induced hypermutations also contribute very little to the viral variation compared to mutations that are generated during reverse transcription.

**Table 5:** Summary of analysis to determine sublethal mutagenesis probability for patient sequences predominantly hypermutated at GA sites^1^.

Study	No. of Seqs.	Seq.	Total nt	GG→AG Mtns/ genome^2^		GA→AA Mtns/ genome		G→A Mtns/ genome		Prob. of
				Total	Stops	Total	Stops	Total	Stops	0 Stops^3^
Eyzaguirre et al. 2013	1	Full-length	9,244	64	12	209	10	273	22	3 × 10^−10^
Gandhi et al. 2008	5	Env/nef	2,282	70	13	167	8	237	22	3 × 10^−10^
Ho et al. 2013	11	Gag, Env, Nef-LTR	11,460	86	17	209	10	295	27	2 × 10^−12^
Total	17		22,986	1,358	262	3,346	161	4,703	422	
Average^4^	-		-	80	15	197	9	277	25	1 × 10^−11^

^1^ Details of the sequence analysis are described in Tables B and D in S1 File,

^2^ Mutations/genome were estimated based on sequence length and average frequency of mutations in the same region of the genome in 11 full-length hypermutated sequences reported by Eyzaguirre et al. [69] to adjust the mutation frequencies for the 5’-to-3’ hypermutation twin gradient [49, 79–81].

^3^ The probability of sublethal mutagenesis, defined as the probability of generating a viral genome without stop codons, was determined by using the average number of stop codon mutations/genome and Poisson distribution.

^4^ The average number of mutations per genome was determined by summing up the total number of mutations estimated per genome for all sequences and dividing by the number of sequences.

### Estimation of the frequency of sublethal mutagenesis by A3G and A3F

To determine the frequency of sublethal mutagenesis by A3 proteins, we analyzed hypermutated proviral DNA sequences reported in three previous studies. Eyzaguire and colleagues reported 11 near-full-length sequences that were hypermutated throughout the proviral genomes [69]. For 10 of the 11 proviruses, the majority of the G-to-A mutations were in the GG context (A3G type); for one provirus, the majority of the mutations were in the GA dinucleotide context (A3F type) and were likely mutated by A3F, A3D or A3H. Gandhi and colleagues reported 166 hypermutated proviral DNA sequences, of which 161 had a majority of the mutations in the GG context (A3G type) and 5 had mutations primarily in the GA context (A3F type) [43]. Ho and colleagues reported 34 hypermutated proviral DNA sequences, three of which were near-full-length and others were from various regions of the genome [78]. Of these, 23 were predominantly mutated in the GG context (A3G type) and 11 were predominantly mutated in the GA context (A3F type).

We estimated the number of G-to-A changes that arose in each proviral genome, taking into account the lengths of the sequences analyzed as well as their locations in the genome. Furthermore, the sequences analyzed were from patients from whom a consensus sequence for the patient could be derived to further verify that the sequence was indeed hypermutated. For our analysis, proviruses with less than 18 G-to-A mutations would not be identified as hypermutants (Table A in S1 File). It is well known that there is a twin-gradient of hypermutation in the viral genome, which reflects the amount of time the minus-strand DNA is available as a substrate for cytidine deamination by A3 proteins [49, 79–81]. We used the 11 near-full-length hypermutated genomes reported by Eyzaguire et al. [69] to estimate the relative frequency of hypermutation for each region of the genome to adjust for the hypermutation gradient. The average number of GG-to-AG mutations estimated per proviral genome was 231 (Table 4 and Tables A and C in S1 File); the sequences that were predominantly hypermutated at GG sites also had an average of 37 mutations at GA sites/genome.

Since 19.3% of the GG sites generated stop codons in NL4-3, we estimated that mutations at GG sites generated an average of 45 stop codons/genome; since 4.8% of the GA sites in NL4-3 generated stop codons, we estimated that an average of 2 stop codons/genome were generated by GA-to-AA mutations, resulting in an average of 47 stop codons/genome. Assuming a Poisson distribution, we estimated that the probability of generating a provirus without stop codons is 4 × 10^−21^. Thus, we conclude that the contribution of A3G-induced sublethally mutated proviruses to viral genetic variation is negligible.

Similarly, proviruses that were predominantly mutated at GA sites had an average of 197 GA-to-AA mutations/genome, and 80 GG-to-AG mutations/genome with an average of 25 stop codons/genome (Table 5 and Tables B and D in S1 File); assuming a Poisson distribution, the probability of generating a sublethally mutated provirus is 1 × 10^−11^. Thus, we conclude that while sublethal mutagenesis can occur, there is a simultaneous overwhelming reduction in the size of the replicating viral population. In the absence of A3F-induced hypermutation, RT and RNA polymerase II would generate 3 × 10^10^ mutations in 1 × 10^11^ proviruses, whereas A3F hypermutation would generate 277 mutations in a sublethally mutated provirus.

## Discussion

**Fig 5:**
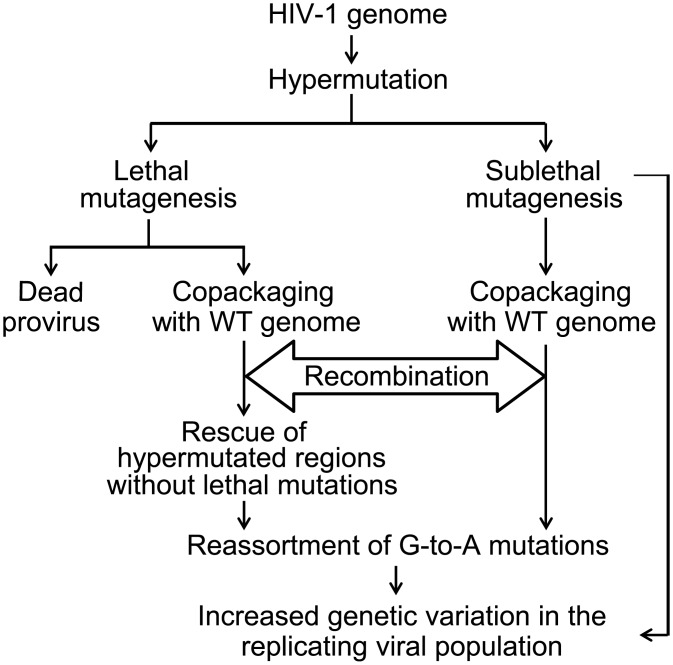
Flow chart of potential contribution of hypermutation and recombination to HIV-1 genetic diversity.

In addition to error-prone reverse transcription and high rates of recombination, G-to-A hypermutation by A3 proteins could increase genetic variation in HIV-1 populations by two mechanisms (Fig 5). First, lethal mutagenesis could generate dead proviruses that cannot increase genetic variation of the replicating viral population; however, parts of such genomes may be rescued when a replication-competent virus infects the same cell through copackaging and recombination. If the resulting recombinants contain portions of hypermutated genomes without lethal mutations, these G-to-A hypermutations can enter the replicating viral population. Second, sublethally mutated viruses can on their own increase genetic variation of the replicating viral population, and through recombination with wild-type genomes, further increase genetic variation. However, hypermutation could also decrease genetic variation by reducing the rate of recombination due to decreased homology between the co-packaged RNAs.

### Effect of hypermutation on recombination

To our knowledge, this is the first study to directly examine the effects of hypermutation on the frequency of retroviral recombination and quantify the extent to which recombination can rescue portions of hypermutated genomes without lethal mutations. In our system, hypermutation by A3G or A3F did not affect the efficiency of RNA copackaging or the frequency of recombination as determined by the rate of *gfp* reconstitution. We also did not observe a decrease in the frequency of recombination in the A3G or A3F hypermutated regions of the *pol* gene (Table 2 and Fig 2). Our previously proposed dynamic copy-choice mechanism of recombination and results from other studies predict that a decrease in homology between co-packaged RNAs would reduce the frequency of recombination [82–86]. The recombination rate was similar in the absence or presence of hypermutation, which decreased the sequence identity in RT to 92–97%. It is possible that more extensive hypermutation (> 64 mutations/1320 nt of RT) would lead to a reduction in the recombination rate. However, hypermutated full-length patient sequences had on average 42 G-to-A mutations in RT [69], compared to 64 for N-A3Ghigh, suggesting that the majority of hypermutated genomes will not affect the rate of recombination.

### Effect of hypermutation on increasing genetic variation through recombination

Our results show that within the population of viruses containing a wild-type and a A3G- or A3F-hypermutated genome, recombination contributed to the retroviral mutation rate to the same extent as mutations during error-prone viral replication (3.9 × 10^−5^ versus 3.4 × 10^−5^ mutations/bp/replication cycle, respectively). However, we expect that because of purifying selection on hypermutated genomes, only a small proportion of hypermutated genomes will be packaged into virions [68]. Additionally, it was recently found that, contrary to a previous report [87], >90% of HIV-1 infected CD4^+^ T cells in lymph nodes of patients contained only one proviral DNA [88], implying that the frequency of copackaging of RNAs from two different proviruses, including RNAs from a wild-type and a hypermutated genome, is likely to be very low [89]. Consistent with purifying selection, Kieffer et al. observed that >9% of the proviral DNAs were hypermutated by A3G or A3F but none of the 2024 viral RNAs isolated from plasma were hypermutated [45]. Since the frequency of copackaging and recombination between hypermutated and wild-type genomes in patients appears to be extremely low (<1/100 –<1/2024; [44, 45, 76, 77]), we conclude that the contribution of hypermutation to viral genetic variation through recombination is far less than mutations that occur during retroviral replication.

Mulder et al. reported that recombination between hypermutated and wild-type genomes resulted in increased resistance to antiviral drug 3TC through acquisition of M184I mutation in RT [65]. In this study, the proviral DNAs underwent one round of DNA transfection (plasmids containing hypermutated genomes and wild-type genomes were co-transfected into cells). DNA recombination is known to occur frequently during co-transfection and has been shown to be sufficient to reconstitute replication-competent retroviruses from defective genomes even in the absence of retroviral recombination [89, 90]. Kim et al. also reported that hypermutation in a T cell line can contribute to selection of M184I mutations and 3TC resistance [66]. It is not clear whether the observed frequencies of M184I mutations (0 of 4 for A3G- cells vs. 3 of 4 for A3G+ cells) are significantly different from each other. It is also possible that, in an experimental system when a limited number of cells and high titers of infectious virus are used, coinfection and recombination occurs at a much higher frequency than in patients, resulting in an increase in 3TC resistance in the presence of A3G. In our studies, we ruled out potential contribution of DNA recombination, and observed the effect of A3G and A3F hypermutation on the viral mutation rate in the absence of selection, which could explain the modest two-fold contribution of hypermutation to the retroviral mutation rate in the progeny from heterozygous viruses.

A recent study determined the HIV-1 mutation rate *in vivo* by determining the frequency of stop-codon mutations in proviral DNAs in patients [91]. As expected, their analysis included all G-to-A mutations induced by A3 proteins, and concluded that the HIV-1 mutation rate in proviral DNA is extremely high (4.1 x 10^−3^/bp/cell), and most of the mutations are due to cytidine deaminase activity of A3 proteins. As our analysis points out, most of the hypermutated proviruses are lethally mutated and cannot contribute to the genetic variation of the replicating viral population. Therefore, we have focused our analysis on the potential contribution of hypermutation to genetic variation on which selective forces can act to shape viral evolution.

Our *in silico* modeling indicated that the number of template-switching events did not affect the contribution of A3G or A3F hypermutation to the retroviral mutation rate. The modeling results indicated that for A3G and A3F, the optimal number of G-to-A mutations (10–15 and 30–35, respectively) would increase the retroviral mutation rate by 3- or 16-fold, respectively, in the population of heterozygous virions. The average numbers of mutations at GG and GA sites in patients (231 and 197, respectively) are much higher than the optimal number of mutations, and their contribution to the retroviral mutation rates are 200-fold lower (7.8 × 10^−7^ mutations/bp/cycle) or about the same (5 × 10^−5^/bp/cycle) as the retroviral mutation rate (3.4 × 10^−5^/bp/cycle), respectively. Even with the optimal number of mutations, given the low frequency of copackaging and recombination (<1/100–<1/2000; [44, 45, 76, 77]), the contribution of hypermutation to the retroviral mutation rate is likely to be far less than the mutation rate during error-prone replication.

### Sublethal hypermutation and its effect on HIV-1 genetic variation

Our analysis of hypermutated proviruses reported in three independent studies [43, 69, 78] predicted that proviruses hypermutated predominantly at GG sites or predominantly at GA sites have on average 47 and 25 stop codons, respectively. Based on a Poisson distribution, the frequency of sublethally mutated proviruses predominantly mutated at GG sites (A3G type) and GA sites (A3F type) is predicted to be 4 × 10^−21^ and 1 × 10^−11^, respectively. Thus, the vast majority of hypermutation events result in lethal mutagenesis, and very few result in sublethal mutagenesis that can potentially increase genetic variation in the replicating viral population. It is important to point out that these are conservative estimates of sublethal mutagenesis, since nonsynonymous G-to-A mutations, as well as some mutations in the cis-acting viral sequences, also likely result in loss of fitness. Even in the absence of stop codons, the hypermutated viruses with many non-synonymous G-to-A mutations are likely to be highly attenuated in their replication potential, further reducing their capacity to contribute to genetic variation. Therefore, the recombinant viruses containing sublethal mutations are also unlikely to outgrow the nonhypermutated parental viruses, and their contribution to genetic variation will likely diminish with each successive replication cycle.

Simon et al. found Vif alleles in patients that were defective in inducing degradation of A3G or A3F proteins, suggesting that incomplete degradation of A3 proteins could lead to sublethal mutagenesis [92]. Sadler et al. observed that expression of lower amounts of A3G resulted in sublethal mutagenesis in a cell culture system, indicating that sublethal mutagenesis can occur in an ex vivo assay [58]. On the other hand, Armitage et al. found that packaging of single active A3G protein in virions results in substantial levels of hypermutation, and concluded that hypermutation by A3G is typically an all-or-nothing phenomenon [64].

Our analysis does not exclude the possibility of sublethal mutagenesis, but implies that such low levels of hypermutation are likely to be rare. One caveat to our studies is that proviral genomes that are identified as hypermutated genomes requires that the regions sequenced need to have at least two G-to-A mutations in order to be defined as hypermutants; sequences with one G-to-A mutation/~450 nt (the length of *env* sequence analyzed in [43]) will not be defined as hypermutants, suggesting a lower limit for detection of hypermutation as ~18 G-to-A mutations/proviral genome (Table A in S1 File).

### Potential contribution of hypermutation to viral genetic variation and evolution

Many previous studies have concluded that A3 proteins contribute to viral genetic variation and evolution [58, 65, 66, 92–96], while others have concluded that hypermutation by A3 proteins does not increase genetic diversity or contribute to viral evolution [60, 62, 64, 97].

A few studies have analyzed the context in which A3-proteins induce mutations and sought to determine whether mutations in these contexts may have provided a selective advantage to the virus, and thereby contributed to viral evolution [60, 66, 94, 98]. One confounding factor in these analyses is that the contexts in which RT and RNA polymerase II induce mutations are not well defined, and the extent to which error-prone viral replication can induce errors in A3-favored contexts is unknown. HIV-1 RT has a strong bias for inducing G-to-A mutations during replication, with nearly 40% of the substitutions occurring in GA context and GG contexts [2, 4]. Additional studies to define the nucleotide contexts of mutations induced by RT and RNA polymerase II are needed to facilitate these analyses.

In summary, we found that A3G or A3F hypermutation did not affect the rate of recombination and the contribution of A3G and A3F hypermutation to the genetic variation of HIV-1 was significantly less than the rate of mutations induced during error-prone viral replication. Thus, while hypermutation can alter sequences in some proviruses, its contribution to viral variation and evolution is small compared to mutations induced by RT and/or RNA polymerase II.

## Material and Methods

### Construction of plasmids containing wild-type and hypermutated RT sequences

The names of all plasmids in this study start with ‘p” while the names of viruses and proviruses generated from these plasmids do not. pHCMV-G that expresses the G glycoprotein of vesticular stomatitis virus (VSV-G) [99], pSYNGP that expresses a codon-optimized HIV-1 Gag/Gag-Pol [100], and pIIINL(AD8)env that expresses the HIV-1 CCR5-tropic envelope [70] have been described previously.

To create plasmid pWT_HXB2_ containing a wild-type *pol* sequence from the subtype B HXB2 isolate, the region between BamHI and XhoI restriction sites in plasmid pHG(B_HXB_) [86] was replaced with a corresponding region from plasmid pON-H0 [13]. The resulting construct pWT_HXB2_ contains all *cis*-acting elements necessary for virus expression and production, functional *gag-pol*, as well as *hsa* and inactivated *gfp* in the *nef* gene [13].

The four constructs containing NL4-3-based RT sequences were created as follows. First, SphI and MscI was used to digest NL4-3-based plasmid pON-T6 [13], which in *nef* contains *thy1*.*2* and IRES followed by an inactivated *gfp* gene. The distance between the inactivating mutations in the two *gfp* genes is 588 bp. To create construct pN-A3Ghigh, the SphI-MscI digested pON-T6 backbone was ligated to a synthesized SphI to MscI fragment (GENEWIZ) that contains the following modifications to NL4-3 sequence: 1) a frameshift and a stop codon was introduced to destroy SpeI site in *gag*; 2) the natural MscI in *gag* was destroyed by a silent mutation; 3) unique enzyme sites SgrAI, SnaBI, and XbaI flanking the start of RT, RNase H and IN, respectively, were introduced via silent mutations; and 4) RT region contained 64 G-to-A hypermutations by A3G that were previously isolated from an infected cell clone. To create pWT_NL43_, pN-A3Glow, and pN-A3F, plasmid pN-A3Ghigh was digested with SgrAI and SnaBI, and ligated with a synthesized insert (GENEWIZ) containing either a wild-type RT sequence from pNL4-3 isolate, an NL4-3 RT sequence with 27 G-to-A changes introduced by A3G, or an NL4-3 RT sequence with 27 G-to-A changes introduced by A3F, respectively. All plasmids were verified by sequencing (Macrogen).

### Generation, maintenance, and flow cytometry analyses of cell lines

Human embryonic kidney 293T cells and derivatives (American Type Culture Collection) were maintained in Dulbecco’s modified Eagle’s medium (CellGro) supplemented with 10% fetal calf serum (HyClone), and 1% penicillin-streptomycin stock (penicillin 50 U/ml and streptomycin 50 μg/ml, final concentration; Gibco). Hut/CCR5 cells, a human T cell line derived from Hut78 cells to express CCR5 chemokine receptor [101], were maintained in RPMI medium (CellGro) supplemented with 10% fetal calf serum (HyClone), 1% penicillin-streptomycin stock (penicillin 50 U/ml and streptomycin 50 μg/ml final concentration; Gibco), 1 μg/ml of puromycin (Gibco) and 500 μg/ml G418 (ThermoFisher Scientific). All cultured cells were maintained in humidified 37°C incubators with 5% CO_2_. All transfections were performed using LT1 reagent (Mirus) according to manufacturer’s instructions.

To detect marker gene expression, cells were stained with phycoerythrin-conjugated α-HSA antibody (Becton Dickinson Biosciences) and allophycocyanin-conjugated α-Thy1.2 antibody (eBioscience) at 0.4 μg/ml and 2.0 μg/ml, respectively. Flow cytometry analyses were performed on a FACSCalibur system (BD Biosciences) whereas cell sorting was performed on an ARIA II system (BD Biosciences). Flow cytometry data was analyzed using FlowJo software (Tree Star).

Producer cell lines containing two different proviruses were generated as follows. To generate stock viruses for infection, 293T cells were transfected with viral construct along with plasmids pSYNGP and pHCMV-G that express codon-optimized Gag/GagPol and VSV-G envelope, respectively. Viruses were harvested 48 hours later, filtered with 0.45- μM filters, and used immediately or stored at –80°C. To make producer cell lines, stock virus WT_HXB2_ was used to infect fresh 293T cells at a multiplicity of infection (MOI) of 0.1. Cells were stained 72 hours post-infection and infected cells expressing HSA surface marker were enriched by multiple rounds of cell sorting until more than 80% of the cells were HSA^+^. These cells were then infected at an MOI of <0.1 with a second virus, and underwent multiple rounds of cell sorting until >97% of cells were HSA^+^ and Thy^+^. Four cell lines were created containing the following pairs of proviruses: WT_HXB2_/WT_NL43_, WT_HXB2_/N-A3Ghigh, WT_HXB2_/N-A3Glow, and WT_HXB2_/N-A3F.

### Recombination experiments and sorting of recombinants

Producer cell lines WT_HXB2_/WT_NL43_, WT_HXB2_/N-A3Ghigh, WT_HXB2_/N-A3Glow, and WT_HXB2_/N-A3F were transfected with pIIINL(AD8)env [70]; 48 hours later viruses were harvested, filtered through 0.45-μM filters, and used to infect 12 × 10^6^ Hut/CCR5 target cells at a low MOI (<0.08) to minimize dual infection. Target cells were stained 72 hours post-infection for marker expression and flow cytometry analysis was used to determine the percentage of HSA, Thy and GFP expressing cells. Target cells expressing GFP were enriched by sorting until 87% of the cells were GFP^+^.

### Viral DNA isolation, single genome sequencing and sequence analysis

Genomic DNA was isolated from the sorted GFP^+^ cell pools using QIAamp DNA blood kit (Qiagen). Single genome amplification was achieved by serially diluting genomic DNA in 96-well plates to identify a dilution in which PCR-positive wells constituted less than 30% of the total number of reactions. At this dilution, most wells contain amplicons derived from a single DNA molecule. PCR amplification was performed in a 20-μl reaction containing 1× High Fidelity Platinum PCR buffer, 2 mM MgSO_4_, 0.2 mM of each deoxynucleoside triphosphate, 0.2 μM of each primer, and 0.025 U/μl Platinum Taq High Fidelity polymerase (Invitrogen). For the first round of PCR, sense primer HIV-A GagF1 5′- GTG GCA AAG AAG GAC ACC TAG-3′ and antisense primer HIV-A VifR1 5′-GTC GAC ACC CAA TTC TGA AAT G-3′ were used. PCR was performed with the following parameters: 1 cycle of 94°C for 2 min, 35 cycles of a denaturing step of 94°C for 15 s, an annealing step of 55°C for 30 s, and an extension step of 68°C for 4 min, followed by a final extension of 68°C for 10 min. For the second round of PCR, we used 1 μl of first-round PCR product along with sense primer HIV-A GagF2 5′- GGC TGT TGG AAA TGT GGA AAGG-3′ and antisense primer HIV-A VifR2 5′- ATG GCT TCC AAT CCC ATA TGA TG-3′. The second-round PCR reaction was performed under the same conditions used for first-round PCR, but with a total of 45 cycles. All PCR procedures were performed under PCR clean room conditions with additional procedural safeguards against sample contamination, such as prealiquoting of all reagents, use of dedicated equipment, and physical separation of sample processing from pre- and post-PCR amplification steps. Correctly sized amplicons from the second round of PCR were sequenced directly by cycle-sequencing using BigDye terminator chemistry according to the manufacturer’s recommendations (Applied Biosystems). Individual sequence fragments for each amplicon were assembled and edited using Sequencher (Gene Codes). Individual chromatograms were inspected for the absence of mixed bases at each nucleotide position throughout the entire amplicon; this quality control measure confirmed that the amplicons analyzed were derived from SGS amplification of a single viral template and allowed us to exclude from the analysis amplicons that resulted from PCR-generated in vitro recombination events or Taq polymerase errors. Therefore, the collection of individual sequences obtained via SGS proportionately represents those found in the infected cells.

In order to identify crossover events, we aligned the nucleotide sequences of each genome in a recombinant pair (WT_HXB2_/WT_NL43_, WT_HXB2_/A3Ghigh, WT_HXB2_/A3Glow, or WT_HXB2_/A3F). Using the polymorphic differences between two parental sequences, we identified the locations of crossover events for each recombinant sequence.

### Computer modeling and simulation

A custom in-house MATLAB program was used to estimate the contribution of hypermutation and recombination to the HIV-1 mutation rate in a population of heterozygous virions containing a wild-type genome and a hypermutated genome. The NL4-3 genome was used as the reference (WT) sequence, and the input variables were 1) the number of template switches, 2) the number of G-to-A mutations in the mutated genome, and 3) the number of heterozygous virions containing a wild-type and a mutated genome simulated to undergo one cycle of replication. All GG and GA sites in NL4-3 (GenBank AF324493.2) were identified and the GG-to-AG and GA-to-AA mutations that would generate stop codons in the appropriate open reading frames were determined. NL4-3 has 616 GG sites, of which 119 (19.3%) are predicted to generate stop codons upon mutation to AG. NL4-3 also has 756 GA sites, of which 37 (4.8%) are predicted to generate stop codons upon mutation to AA. The locations of the template switches and G-to-A mutations were randomly selected. For an A3G-mutated genome, we simulated 90% of the mutations at GG sites and 10% at GA sites, and for an A3F-mutated genome we simulated 86% of the mutations at GA sites and 14% at GG sites; these ratios were based on our previously published [68] observations of the G-to-A mutations induced by A3G and A3F in *ex vivo* experiments. The viable recombinants, defined by the absence of mutation-induced stop codons, were identified. The mutation rate was calculated as the total number of mutations in viable recombinants divided by the total number of nucleotides from the viable and nonviable recombinants.

## Supporting Information

S1 File Analysis of G-to-A hypermutation from patient proviral sequences. (Table A) Analysis of patient sequences that were predominantly hypermutated at GG sites. (Table B) Analysis of patient sequences that were predominantly hypermutated at GA sites. (Table C) Calculations used to prepare Table 4. (Table D) Calculations used to prepare Table 5.
(XLSX)
